# The Military Miscellany; Comprehending a History of the Recruiting of the Army, Military Punishments, &c. &c.

**Published:** 1847-01

**Authors:** 


### Art. XII.

*The Military Miscellany; comprehending a History of the Recruiting of the Army, Military Punishments, &c. &c.* By Henry Marshall, F.R.S.E., Deputy Inspector-General of Army Hospitals, &c. &c.—*London,* 1846. 8vo, pp. 375.

Although the volume before us is not, strictly speaking, a professional work, it has many claims to our notice as medical reviewers. While the object of the author is stated to be "to supply the reader with some information respecting the constitution, laws, and usages of the army, and to excite attention to the means which may meliorate the condition of soldiers, and exalt their moral and intellectual character," he has entered at some length into questions of great importance to the army surgeon, and particularly as relates to his duty with reference to military punishments, and to the effects of these on the health of the soldier. Mr. Marshall is already favorably known to the profession by his 'Notes on the Medical Topography of Ceylon,' and his valuable work 'On the Enlisting, Discharging, and Pensioning of Soldiers,' which is still the best in our language on feigned diseases. To him also we owe, in a great measure, the valuable Military Statistical Reports, as he drew up the plan on which they were framed, and for some time superintended the preparation of them. It is not very creditable to the authorities that they permitted the officer who was intrusted with, and satisfactorily performed, this laborious and responsible task to remain upon half-pay without the slightest acknowledgment of his valuable services.

In a motto prefixed to the Miscellany, our author, in the quaint words of Sir James Turner, assigns a reason for having compiled this work: "You ask me, what moved me to write these discourses? If I were put to the rack till I gave you my reason, I could give you no other than this, that being out of employment and not accustomed to an idle life, I knew not how to pass away my solitary and retired hours with a more harmless divertisement." We would be well pleased to see many more members of the profession spend their leisure hours in so praiseworthy a manner.

Our author gives an interesting historical account of the various modes of recruiting the army, from the days when the justices were required "to raise as many men *by impress*, for soldiers, gunners, and *chirurgeons*, as might be appointed by His Majesty and both Houses of Parliament," till the present time, when an error in the mode of attesting a recruit has been deemed a sufficient reason to grant him his discharge. But we must pass over these details, interesting and amusing as they are, and turn to that section of the work which more immediately treats of the duty of the medical officer, viz. that on punishments in the army. These may be considered in two points of view: 1st, as regards the duty and responsibility of the military surgeon; and, 2dly, with reference to their effects upon the health and efficiency of the soldier.

I. *The duty and responsibility of the surgeon in cases of corporal punishment in the army.* It is a remarkable fact, that no instructions nor code of regulations of any kind have ever been promulgated by the military authorities for the guidance of the surgeon when called upon in the course of his duty to superintend a punishment. When a soldier is tried by a court-martial, the following certificate is laid before the court:

"I certify that No. ——, Private ——— ———, of the —— regiment, is in a good state of health, and fit to undergo corporal punishment or imprisonment, solitary or otherwise, and with or without hard labour."

(*Signature of the Surgeon or Assistant-Surgeon.*)

If he is sentenced to receive corporal punishment, it is necessary, according to the regulations for the army, that a medical officer should be present when it is inflicted. This rule appears to have been framed for a twofold purpose: 1st, to prevent the prisoner escaping any portion of the punishment if able to bear it; and, 2d, to prevent the infliction being carried to such an extent as to endanger life. This has been very clearly stated by Lord Chief Baron Macdonald, on the trial of Colonel Wall, in 1802:

"It is usual," said his lordship, "even in the infliction of ordinary punishments, that the assistance of surgeons should be called in, when the punishment is intended at the outset to be only such as experience shows us is never, without a very singularly unlucky accident, attended with death. The medical officer is, it would appear, to guard the life of a delinquent under punishment, so that the army may not lose the services of a man by death or by being permanently disabled. In the execution of this highly important duty he must be guided by a knowledge of the physiology and pathology of the human body, the habits and duties of soldiers, and an acquaintance with the regulations and usages of the army. A medical officer is presumed to divest himself of any opinion he may entertain in regard to the delinquency a man has committed, or the sentence which has been awarded him; his duty being, in the first place, to prevent the man from escaping punishment by feigning indisposition; and secondly, to see that he does not receive such a degree of injury as may endanger life or disable him permanently for the duties of a soldier. While the surgeon should invariably lean to the side of safety, duty requires that he ought to be scrupulously careful not to unnecessarily obstruct the course of military law—the rules and usages adopted to establish and sustain military discipline."

Our author advises medical officers to recommend that corporal punishments should not in any case be inflicted during the great prevalence of endemic or epidemic disease, or when symptoms of scurvy or ill-conditioned sores appear among the men. We would be inclined to extend this to periods when the weather is either unusually hot or very cold.

"The medical officer usually takes his station a few paces behind the man who is undergoing punishment; but, should symptoms of fainting come on, he sometimes moves towards the front of the sufferer, so as to see his face." (p. 273.) "Pain, but especially pain which is inflicted or imposed as a chastisement, frequently excites fainting, or *deliquium animi,* and when this takes place it becomes highly expedient to arrest the infliction of punishment. . , . . . But a man under punishment is liable to a partial *deliquium animi,* or fainting, during which it has been recommended (and it is, I suppose, usual) to permit the punishment to go on during some seconds of impaired sensibility. In the slighter cases, therefore, of *deliquium* the punishment need not be interrupted; indeed, the stimulus of flagellation frequently restores the sufferer to himself. If, on the other hand, the *deliquium* continues and a man cannot be roused in a few seconds, if he perspires much, and if the pulse at the temporal artery becomes weak or scarcely perceptible, he should be forthwith taken down. I never considered it expedient to examine the irritability of the iris, as is sometimes recommended in doubtful cases, being always satisfied with the conclusions which might be drawn from the above symptoms." (p. 285.) "When an unusual degree of tumefaction of the back takes place during punishment, a delinquent should be taken down, as this symptom is frequently followed by long protracted disease." (p. 290.)

When the surgeon deems a soldier incapable of bearing any further punishment without risk of danger, it is his duty to report this to the commanding officer and to recommend that the man be taken down. We are informed by the Adjutant-General (Report on Military Punishments), "that it is at the *peril* of a commanding officer to order the infliction of a single lash after a medical officer interferes for the purpose of suspending punishment." It has occasionally happened that commanding officers have refused to comply with the suggestion of the surgeon, and have ordered the punishment to proceed; but this is a responsibility which few or none would be inclined to incur in the present day.

It would appear to be the opinion of military officers, that when death ensues, consequent upon flogging, the surgeon who superintends the punishment would be the responsible party. Thus, Major James (Regimental Companion) says, "we cannot omit mentioning in this place, that the instant a military culprit receives a lash, the surgeon becomes responsible for his life." And Sir Charles Napier (On Military Law) observes, "The fact is, that the medical officers are placed in a most unfair and perilous position. The danger to which the life of the culprit and *the life of the surgeon* are exposed, appears to be a powerful objection to this punishment. As to making the surgeon responsible, it is unjust to do so; the law places a man *by force* in a certain position, and orders him to act according to the best of his judgment. He does so, and there is an end of the matter, whatever may be the consequences, unless it can be shown that he was drunk or mad!" With the gallant officer's opinion on the equity of the case we cordially agree, but we differ from him with regard to the legal responsibility. The only recorded opinion of a judge upon this point, so far as we are aware, is that of Lord Chief Baron Macdonald, on the trial of Governor Wall, already alluded to. Referring to the non-interference of the surgeon, he observed: "I think it necessary to tell you that, if a punishment is inflicted unusual in its circumstances, either as to quantity or the instrument with which that punishment is inflicted, it will not take off from those who inflict that punishment a great deal of responsibility. . . . . . Notwithstanding the surgeon attends, and notwithstanding he does not interfere and make representations upon it, they who inflict the punishment, if it should be most inordinate in its quantity, or in the manner of inflicting it, by the nature of the instrument or otherwise, may, under certain circumstances, not exculpate themselves." (p. 275.) Paris and Fonblanque state, that "it is generally supposed the surgeon who is present at a military execution is responsible for its consequences; *this is not legally true,* and it is physiologically impossible: the punishment is too uncertain in its operation to allow of any medical officer ascertaining the boundaries of danger."

We do not know what view the authorities at the Horse Guards might take in such a case, but we believe that, unless culpable negligence were proved, he would be acquitted by a civil tribunal.

II. *Influence of punishment on the health and efficiency of the soldier.* In discussing the different kinds of military punishments, Mr. Marshall proclaims himself an uncompromising enemy to flogging. We unhesitatingly rank ourselves by his side, and denounce it as a degrading and demoralizing practice, which *ought to be* unjustifiable in the case of a person in the position of a soldier. The great difficulty, however, is to find an adequate substitute for it, as no one can deny that, in the actual state of society, and with the amount of intellectual and moral cultivation possessed by men in the rank of our soldiery, some kind of punishment is absolutely necessary for the support of military discipline and efficiency. At present we have only to judge between the practice of *flogging* and the practice of *solitary imprisonment;* and looking at them both impartially, we are forced to come to the conclusion, that of the two, *as at present inflicted,* the former is preferable to the latter—that is to say, that a slight flogging is less injurious, both physically and morally, than a severe imprisonment. Surely the time is not far distant when both will be dispensed with, and some other mode of enforcing the requisite degree of order be discovered, less injurious to both body and mind. But assuming, for the present, that punishment is necessary, and that we have only to decide between the two in question, let us briefly consider their respective advantages and disadvantages. In the following remarks, it must, of course, be borne in mind that we do not at all advert to the system which was pursued when our author entered the service, whereby a court-martial had the power to sentence the prisoner to receive an unlimited number of lashes: one instance is on record of 1900 having been adjudged, and several of 1000 having been inflicted. In the present day the utmost number permitted by the Mutiny Act is 200, and even that has been further limited by a recent order of the Commander-in-chief, to 50.

The objections of our author to corporal punishment may, we think, be fairly summed up under the following heads: 1, it is cruel and inhumane; 2, it is very unequal; 3, it is ineffectual as an example to deter from crime; 4, it has no tendency to reform the culprit; 5, it leaves an indelible mark. All punishment, it must be admitted, is an evil; but it is, or ought to be, the infliction of a less evil for the prevention of a greater. For the prevention of crime and the maintenance of discipline in the army, we fear some sort of punishment will be found necessary till that peaceful period arrives when nations shall study war no more. The question, therefore, appears to be not as to the existence but the nature of the punishment.

For the more serious offences in the army, not involving sentence of death or transportation, there are, as we have said, only two kinds of punishment—flogging and imprisonment. Let us briefly examine whether the objections of our author to the former are not equally, or even more applicable to the latter. 1. On the ground of cruelty and inhumanity. We have often seen soldiers discharged from prison on the expiry of their sentence, broken down in health and spirits, and scarcely fit for duty afterwards. Now we maintain there is less cruelty or inhumanity in flogging a man, especially under the restrictions now in force, than in inflicting a punishment, the result of which may very possibly be to shorten his life, or induce such a state of health as to necessitate his discharge from the army without a pension, without health to work for his daily bread, and with the prospect of lingering out a miserable existence as the inmate of a union workhouse. 2. The objection that it is unequal in its effects applies also to imprisonment. This is fully corroborated by a quotation from the late Dr. Malcomson (Letter to Sir H. Hardinge), whose opinion is entitled to great weight:

"Many men, particularly those of indolent habits, endure a confinement of four or six weeks on bread and water without injury to their health; but in some instances, a shorter period is sufficient to cause a total loss of appetite; the bread is hardly touched, and no other food being allowed, the patient is unable to eat or to digest it." (p. 300.)

3. That it is ineffectual as an example to deter from crime is, in our opinion, much more true as applied to imprisonment than flogging. In the one case, the infliction of the punishment is witnessed by the man's comrades, in the other, it is undergone at a distance, and the severity of its character is matter of speculation. 4. Flogging has no tendency to reform the culprit. We freely admit this; but we must add that we never yet saw a soldier morally improved by any length of imprisonment. Indeed, under the old system, when they were incarcerated along with civil criminals, the reverse was too often the case, and the acquaintances formed in prison were of a nature which tended to anything but improvement. During the last twelve months military prisons have been established throughout the kingdom, and this source of moral contamination has been removed. *Omne ignotum pro magnifico* is a principle so universal in its application, that we confidently anticipate hearing much of the beneficial effects of these new establishments, but we much fear time will prove their reformatory influence to be vastly overrated. 5. The objection of its leaving an indelible brand will not, we believe, apply to the present reduced amount of punishment.

It would thus appear that the objections to flogging, except the last, are equally, or more applicable to imprisonment; but the latter is liable to two very serious ones from which the former is free. 1st. Its deteriorating effect on the health, especially when of any duration; and, 2d, its injustice to the well-conducted soldier, who is obliged to do extra duty during the period his comrade is in prison. This last is a very serious objection, which, we think, has not received due consideration from the advocates for the abolition of flogging; it is, in fact, extending a species of indulgence to the man of bad character at the expense of the well-conducted soldier. But the fact is even more forcible, as it involves a permanent injury to the individual and an ultimate loss to the service. The effect of imprisonment on the bodily and mental powers does not appear to have obtained the consideration it deserves; indeed, the only work on the subject deserving of mention is the admirable paper by Dr. Baly, in the 28th vol. of the 'Medico-Chirurgical Transactions.' Mr. Marshall has given several extracts from the Reports of the Inspectors of Prisons which fully bear out our observations (p. 300). These evils may perhaps be attributable, in some measure, to the reduced diet on which prisoners are placed as part of their punishment. As regards the present system, our author remarks:

"By the rules for the District Military Prisons, the diet is ordered to be for prisoners not in solitary confinement, 12 oz. of oatmeal or bread with half a pint of milk for breakfast, and 5 lbs. of potatoes with a pint of milk for dinner; if in solitary confinement, by sentence of a court-martial, 10 oz. of oatmeal or bread with half a pint of milk, and 4 lbs. of potatoes with a pint of milk; and if in solitary confinement for a prison offence, 1 lb. of bread, daily, with water for drinking *ad libitum;* but this punishment must not continue longer than seventy-two hours at a time for the reasons already stated. I do not consider this an adequate diet, and it seems doubtful whether the Secretary at War consulted any medical officer having much experience of *military* prisoners, before adopting this scale. It would be out of place here to dilate further upon the evils of an insufficient and inadequately varied diet, added to confinement, want of exercise, and depressing passions. It is sufficient to state that they are calculated greatly to injure the constitution, and to excite the most formidable diseases, although from their anomalous character, these often escape detection until too late to be remedied by art. When the health becomes impaired by scanty nourishment, the subsequent addition to the diet may fail to restore it."

From a full consideration of the question we are of opinion that corporal punishment, as at present restricted, is preferable to imprisonment in the army, because it is less injurious to the individual, it is more efficacious in deterring from subsequent crime, and as a warning to others, and it prevents a great injustice towards the well-behaved soldier. If the condition of the soldier could be so much improved as to render dismissal from the army a severe punishment, then might flogging and imprisonment both be abolished, but this is a desirable event which we fear it will never be our good luck to see accomplished.

The commissioners appointed to inquire into the subject of punishments in the army, and on whose report the system of military prisons was adopted, put the following question to the witnesses: "Have you observed the habits of the released men to be materially altered or *their health essentially affected?*" The answers to this exhibit a considerable difference of opinion, but it is worthy of remark, that not a single medical officer in the army was called before the commissioners. The report, therefore, contains ths *opinions* of military officers from the rank of Major-General to that of Captain, but the regimental surgeons, who from their position and their duty, must have the best means of judging on this point, were not called upon for the result of their experience! We feel confident their information on this subject would have been found much more accurate and trustworthy.

While we differ thus materially from Mr. Marshall in our estimate of the relative effects of corporal punishment and imprisonment, we most cordially agree with him in his desire to see the prevention of crime the great object of attention. The increase of education in quality as well as quantity, the introduction of healthy amusements to counterbalance the attractions of the canteen, the improvement of the barrack-rooms, thereby affording the soldier a comfortable home in the evening, and a general increased attention to his wants and comfort, would tend more to reduce the necessity for punishment than any coercive measures. The army owes a debt of gratitude to our author for having exposed many abuses, and called attention to the means of ameliorating the soldier's lot. We strongly recommend the work to the perusal of our readers as instructive and amusing, containing much interesting matter on hygiene, and characterized throughout by a kindly feeling towards the soldier.

On one point connected with this subject we desiderate more accurate information, the influence of imprisonment on the soldier's health; and we sincerely trust the Head of the Army Medical Department will call for this from the surgeons in charge of the military prisons, and, should the result be as we anticipate, fearlessly bring the matter under the notice of the authorities.

---

